# Chloride Channel Family in the Euhalophyte *Suaeda altissima* (L.) Pall: Cloning of Novel Members SaCLCa2 and SaCLCc2, General Characterization of the Family

**DOI:** 10.3390/ijms24020941

**Published:** 2023-01-04

**Authors:** Olga I. Nedelyaeva, Larissa G. Popova, Dmitrii E. Khramov, Vadim S. Volkov, Yurii V. Balnokin

**Affiliations:** K.A. Timiryazev Institute of Plant Physiology RAS, Moscow 127276, Russia

**Keywords:** *Suaeda altissima*, anion transporter, chloride channel family, euhalophyte, *SaCLCa2*, *SaCLCc2*

## Abstract

CLC family genes, comprising anion channels and anion/H^+^ antiporters, are widely represented in nearly all prokaryotes and eukaryotes. CLC proteins carry out a plethora of functions at the cellular level. Here the coding sequences of the *SaCLCa2* and *SaCLCc2* genes, homologous to *Arabidopsis thaliana CLCa* and *CLCc*, were cloned from the euhalophyte *Suaeda altissima* (L.) Pall. Both the genes cloned belong to the *CLC* family as supported by the presence of the key conserved motifs and glutamates inherent for CLC proteins. SaCLCa2 and SaCLCc2 were heterologously expressed in *Saccharomyces cerevisiae GEF1* disrupted strain, *Δgef1*, where *GEF1* encodes the only CLC family protein, the Cl^−^ transporter Gef1p, in undisrupted strains of yeast. The *Δgef1* strain is characterized by inability to grow on YPD yeast medium containing Mn^2+^ ions. Expression of *SaCLCa2* in *Δgef1* cells growing on this medium did not rescue the growth defect phenotype of the mutant. However, a partial growth restoration occurred when the *Δgef1* strain was transformed by *SaCLCa2*(C544T), the gene encoding protein in which proline, specific for nitrate, was replaced with serine, specific for chloride, in the selectivity filter. Unlike *SaCLCa2*, expression of *SaCLCc2* in *Δgef1* resulted in a partial growth restoration under these conditions. Analysis of *SaCLCa2* and *SaCLCc2* expression in the euhalophyte *Suaeda altissima* (L.) Pall by quantitative real-time PCR (qRT-PCR) under different growth conditions demonstrated stimulation of *SaCLCa2* expression by nitrate and stimulation of *SaCLCc2* expression by chloride. The results of yeast complementation assay, the presence of both the “gating” and “proton” glutamates in aa sequences of both the proteins, as well results of the gene expression in euhalophyte *Suaeda altissima* (L.) Pall suggest that SaCLCa2 and SaCLCc2 function as anion/H^+^ antiporters with nitrate and chloride specificities, respectively. The general bioinformatic overview of seven *CLC* genes cloned from euhalophyte *Suaeda altissima* is given, together with results on their expression in roots and leaves under different levels of salinity.

## 1. Introduction

Genus *Suaeda* related to *Amaranthaceae/Chenopodiaceae* includes more than 100 species of herbaceous highly salt tolerant plants known as salt accumulating halophytes (euhalophytes) [1,2]. Various aspects of plant responses to salt stress are under investigation with the use of *Suaeda* plants at molecular-genetic and functional levels [3,4,5,6,7,8,9]. Some representatives of this genus such as *S. salsa*, *S. maritima*, *S. fruticosa*, *S. altissima*, and others have become good models for investigation of mechanisms underlying salt tolerance, particularly ionic regulation and compartmentation at the cell and whole plant levels, osmotic adjustment through biosynthesis of compatible solutes, antioxidant capacity regulation, transition to C_4_- and CAM-photosynthetic metabolism and others [3,4,5,6,7,8]. Intensively developing transcriptomics, genomics and proteomics in combination with functional studies of *Suaeda* plants are becoming a promising approach in developing strategies for increasing crop resistance to salt stress [9].

One of the most important aspects of plant salt tolerance are ion relations allowing, under salinity, the maintenance of Na^+^ and Cl^−^ concentrations in cytoplasm at nontoxic levels as well as holding mineral nutrition and water balance within tolerable limits. Halophytes, plants of saline habitats, have evolved mechanisms to successfully meet the challenges of saline environments [7,10,11,12]. Transport of anions, Cl^−^ and NO_3_^−^, in plants under salt stress conditions is investigated to a much lesser extent than transport of cations Na^+^ and K^+^ [12,13,14,15,16]. Very few reports are devoted to anion transport and anion transporting proteins in halophytes although these issues are principal for understanding mechanisms underlying plant salt tolerance and its improvement in crops by genetic engineering methods.

Proteins of the CLC (ChLoride Channel) family are found in representatives of all kingdoms [17,18,19,20,21] and play important roles in the transport of Cl^−^ and NO_3_^−^ in plants [20,22]. Contrary to animal CLCs, all of the plant CLCs whose subcellular localization has been studied have been found so far in intracellular membranes, including the tonoplast [20,22,23]. The plant CLC family includes Cl^−^/H^+^- and NO_3_^−^/H^+^-antiporters along with chloride channels [20,23]. The anion/H^+^-antiporters carry out electrogenic exchange transporting anions and H^+^ with the stoichiometry of 2/1 across membranes of cytoplasmic organelles [24,25]. Through Cl^−^/H^+^ exchange, Cl^−^ ions accumulate in the lumen of organelles, including vacuoles; this is one of the key processes responsible for the reduction of Cl^−^ concentration in the cytosol under saline conditions. Along with Cl^−^ transport into the organelles, the Cl^−^/H^+^ antiport stimulates acidification of the organellar lumen through V-ATPase activation mediated by neutralization of positive charges entering the lumen when the ATPase operates [26]. NO_3_^−^/H^+^-antiporter AtCLCa was shown to be involved in NO_3_^−^ storage in vacuoles and regulation of NO_3_^−^ concentrations in the cytosol of *Arabidopsis thaliana* cells [22,27,28]. It was shown that, in glycophytes, salt-sensitive plants, the CLC proteins participated in vesicular trafficking, response to medium salinization, and nitrogen nutrition. Molecular genetic aspects, functions and physiological roles of plant proteins related to the CLC family have been outlined in detail in many publications [20,23,29,30].

In previous studies, we have cloned 5 genes of the *CLC* family from the euhalophyte *Suaeda altissima*: *SaCLCa1*, *SaCLCc1*, *SaCLCd*, *SaCLCf*, and *SaCLCg* [31,32,33], homologs of the *CLCs* from *A. thaliana*: *AtCLCa*, *AtCLCc*, *AtCLCd*, *AtCLCf*, and *AtCLCg*, respectively. Effects of salinity on expression of these genes and ionic specificity of the encoded proteins have been also investigated. The euhalophyte *S. altissima* is one of the most salt tolerant plants and able to perform its life cycle at NaCl concentrations up to 1 M; it does not reduce organ masses at 400 mM NaCl when compared with plants grown in the absence of NaCl in the nutrient solutions (NS). The water potential gradient in (NS—root—shoot) system for *S. altissima* was shown to be positive (the potential values decreased in ascending direction) even if the NaCl concentration in the NS was elevated up to 750 mM. A considerable contribution to maintaining this gradient was made by the accumulation of Na^+^ and Cl^−^ ions in the organs by a gradient manner [5]. It is conceivable that the Cl^−^ accumulation observed in *S. altissima* organs in response to elevation of NaCl concentration in the NS [32] is accomplished by CLC proteins.

In the current work, in addition to the *CLCs* cloned earlier, we report the cloning of two new genes of this family from *S. altissima*, *SaCLCa2* and *SaCLCc2*, the homologs of *AtCLCa* and *AtCLCc*, respectively, and the examination of the anion selectivity of the encoded proteins. The anion selectivity of SaCLCa2 and SaCLCc2 proteins was investigated in complementation tests by heterologous expression of their genes in yeast *Saccharomyces cerevisiae GEF1* disruption mutant, *Δgef1*. *GEF1* is the only gene from the *CLC* family in *S. cerevisiae*, and Gef1p is characterized by Cl^−^ specificity [34]. Expression of *SaCLCa2* and *SaCLCc2* genes in roots and leaves was also studied in *S. altissima* plants subjected or not subjected to NaCl stress and grown at different levels of nitrate availability.

## 2. Results

Coding nucleotide sequences (CDSs) of *SaCLCa2* and *SaCLCc2*, the genes from the halophyte *S. altissima*, were determined based on the putative similarity of these genes to homologous genes from the halophyte *S. fruticosa*. As a result of in silico search of sequences related to the *CLC* family in the de novo assembled transcriptome of *S. fruticosa* [31,35], the contigs containing the coding regions of two sequences homologous to the *A. thaliana CLC* genes were found and designated by us as *SfCLCa2* and *SfCLCc2*. The contigs from *S. fruticosa* served as a base for identification of full-size coding sequences of the target *S. altissima* genes by a rapid amplification of 3’- and 5´-cDNA ends. The cDNAs of the *SaCLCa2* and *SaCLCc2* genes thus obtained were then cloned and sequenced (GenBank, acc. no. OM994378 and GenBank, acc. no. OM994379, accordingly). This approach led earlier to cloning of 5 *CLC* genes from *S. altissima* [31,32,33].

**Figure 1:**
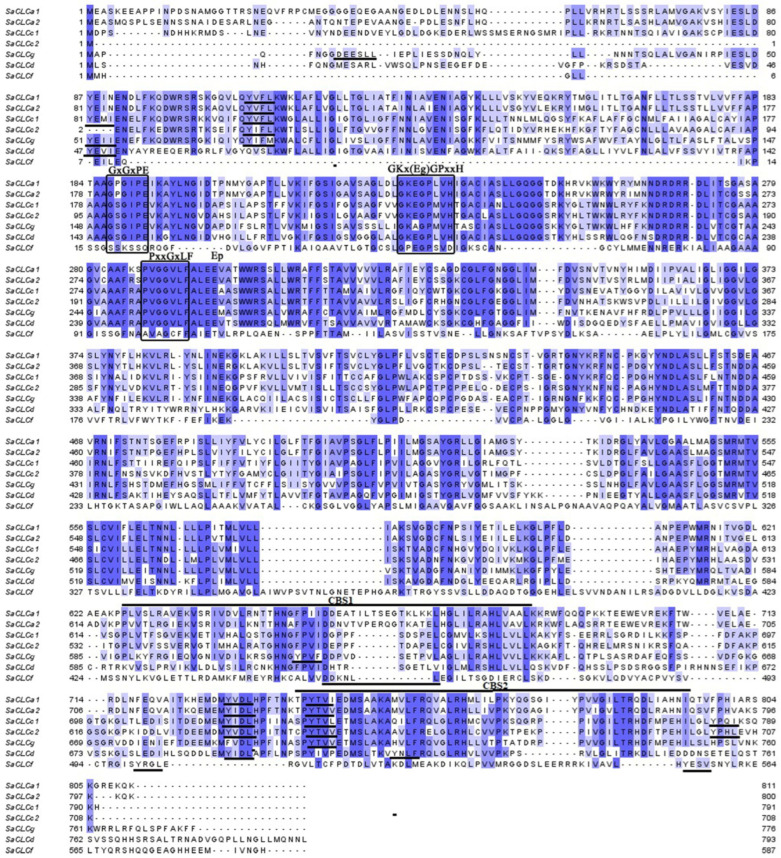
Alignment of the amino acid sequences of CLC proteins from *Suaeda altissima*: SaCLCa1 (GenBank, acc. no. ANG09048.1), SaCLCa2 (GenBank, acc. no. OM994378), SaCLCc1 (GenBank, acc. no. AVQ93350.1), SaCLCc2 (GenBank, acc. no. OM994379), SaCLCd (GenBank, acc. no. OK626332), SaCLCf (GenBank, acc. no. OK626333), and SaCLCg (GenBank, acc. no. OK626334). The alignment was performed in the MAFFT program and visualized in Jalview 2.11.1.4 program. The conserved amino acid motifs (GxGxPE, GKxGPxxH and PxxGxLF) are framed. GxGxPE motif is a selective filter. Eg and Ep are the key glutamates of the CLC family proteins. The intensity of staining for amino acid residues depicts the degree of their identity (identity percentage). Putative sorting signals YXXΦ (where Φ is a bulky hydrophobic residue) and [D/E]XXXL[L/I], CBS1 and CBS2 domains are underlined.

Cloned cDNAs of the *SaCLCa2* and *SaCLCc2* genes contained open reading frames (ORFs) encoding polypeptides consisting of 800 and 708 amino acids, with calculated molecular masses of 88.2 and 77.5 kDa, respectively. Amino acid sequences of the SaCLCa2 and SaCLCc2 were aligned with those of other SaCLC proteins revealed by us previously [31,32,33]. The SaCLCa2 protein is comparable in size with SaCLCa1 (811 a.a.), SaCLCc1 (791 a.a.), SaCLCd (793 a.a.), and SaCLCg (776 a.a.) (Figure 1), as well with AtCLCa (775 a.a.), AtCLCb (780 a.a.) [36], and a protein encoded by a sequence found in *S. fruticosa* transcriptome, *SfCLCa2* (800 a.a.) (Figure S1a). The other protein identified in *S. altissima*, SaCLCc2, is smaller than the above mentioned SaCLCs and its homolog AtCLCc (779 a.a.), but SaCLCc2 is longer than a short isoform of SaCLCf (586 a.a.) (Figure 1 and Figure S1b). The shortened isoforms of CLC proteins have been found in transcriptomes of other plants: *Olea europaea* subsp. *europaea* (CAA 2963713.1), *Olea europaea* var. *sylvestris* (XP_022861637.1), *Phoenix dactylifera* (XP_038975861.1), *Citrus clementina* (XP_006431490.1), *Vitis vinifera* (XP_010657886.1), *Handroanthus impetiginosus* (PIN10391.1), *Eucalyptus grandis* (XP 010030224.1) (Figure S2). Shortening always occurred at the N-terminus of the proteins.

Both proteins identified in *S. altissima* contained three conserved motifs, namely (1) GxGxPE, (2) GKxGPxxH, and (3) PxxGxLF (Figure 1, outlined by rectangles), which are a common feature of all CLC proteins and have been found earlier in animals, yeast, bacteria and *A. thaliana* [29]. In amino acid sequences of the SaCLC proteins, these motifs occupied the positions given in Table S1. These conserved motifs are involved in the formation of the anion-conducting pathway through membranes, in determination of channel ionic selectivity, and in gating of the anion-conducting pathway. The motif GxGxPE functions as a selectivity filter [37]. The amino acid occupying the second position in the motif has been shown to be responsible for the anion specificity of the CLC protein, namely proline (P) for NO_3_^–^ and serine (S) for Cl^−^ [38,39]. The GxGxPE of SaCLCa2 included proline, while the motif in SaCLCc2 contained serine, indicating involvement of the first protein in NO_3_^–^ transport and the second one in Cl^−^ transport (Figure 1 and Table S1). It should be noted that all three conserved amino acid motifs inherent in members of the chloride channel family are found in all identified shortened isoforms (Figure 1, Figures S1b and S2) suggesting involvement of the isoforms in ion transport.

Two conservative glutamates, E1 and E2 (E225 and E293 for SaCLCa2, and E142 and E209 for SaCLCc2), were also found in the amino acid sequences of these proteins (Figure 1, Table 1 and Table S1).

In N- and C-terminal domains located to cytoplasm of SaCLCa2, SaCLCc2, as well as of other SaCLC proteins consensus tyrosine-based motifs YXXΦ (Φ is a hydrophobic amino acid) and dileucine-based motifs DXXLL or [DE]XXXL[LI] were found (Figure 1, underlined). A key role of similar motifs in sorting and subcellular localization was shown for a number of membrane *A. thaliana* proteins [40,41,42,43,44,45].

SaCLCa2, SaCLCc2, and other SaCLCs contain also the regulatory cystathionine beta synthase (CBS) domains CBS1 and CBS2 in the hydrophilic region at the C-terminus (Figure 1 and Figure S2, and Table 1).

**Figure 2:**
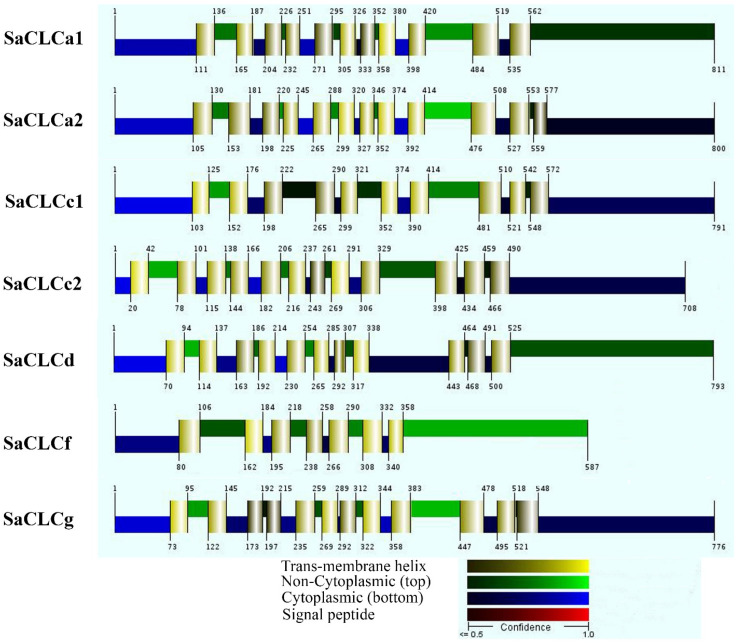
Membrane topology of SaCLC proteins predicted by Philius software (version 3.0, https://noble.gs.washington.edu/proj/philius/; accessed on 15 December 2022).

According to the topology models predicted by the Philius software (accessed on 15 December 2022), SaCLCa2 and SaCLCc2, like other SaCLC proteins, are integral membrane proteins. SaCLCa2 and SaCLCc2 form 12 transmembrane domains with the N- and C-termini located on the cytoplasmic side of the membrane with a stronger confidence prediction for the N-ends and a lesser one for the C-ends (Figure 2).

**Figure 3:**
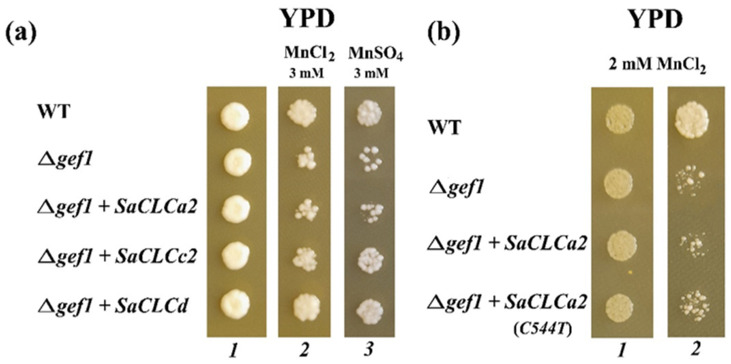
Growth of the yeast mutant *Δgef1*, transformed with *SaCLCa2*, *SaCLCc2* and *SaCLCd* genes. Controls: wild type W3031A and the mutant *Δgef1* transformed with vector pMB1. Approximately 10^5^ cells were placed and grown on selective media. Growth media for (**a**): lane 1—YPD; 2—YPD + 3 mM MnCl_2_; 3—YPD + 3 mM MnSO_4_; Growth media for (**b**): 1—YPD; 2—YPD + 2 mM MnCl_2_; The duration of growth was 3 (**a**) and 2 (**b**) days.

To clarify functions of the SaCLCa2 and SaCLCc2 proteins, we performed heterologous expression of the genes encoding these proteins in the knockout mutant strain of yeast *Saccaromyces serevisiae*, *Δgef1*, produced by us earlier based on the wild-type strain W3031A [31]. In *Δgef1*, the single *CLC* gene in *S*. *cerevisiae*, *GEF1*, encoding Cl^−^ transporter Gef1p, was disrupted. Under the control of the strong constitutive GPD1 promoter, transformation of the mutant strain of *S. cerevisiae Δgef1* was performed by the constructs *pMB1–SaCLCa2*, *pMB1–SaCLCa2*(C544T), *pMB1–SaCLCc2*, and *pMB1–SaCLCd* created on the basis of the shuttle vector pMB1 and containing CDSs of *SaCLCa2*, *SaCLCa2*(C544T), *SaCLCc2*, or *SaCLCd* genes. The *pMB1–SaCLCd* was applied as a positive control, specific for Cl^−^; the growth defect of *Δgef1* was earlier shown to be rescued by expression of the *pMB1–SaCLCd* [33]. To determine the phenotype of the transformants, the strains obtained were plated on the agarized rich medium YPD containing MnCl_2_ or MnSO_4_ at a toxic Mn^2+^ concentration of 2 mM or 3 mM, respectively. In agreement with Gaxiola [46], in our experiments, the growth of the mutant strain *Δgef1* was suppressed by Mn^2+^ ions, unlike the growth of wild type cells and *Δgef1* transformed with *pMB1–SaCLCd*. Expression of *SaCLCa2* did not restore the growth of *Δgef1* colonies on YPD medium containing Mn^2+^ ions (Figure 3a). The inability to restore growth of *Δgef1* by expression of SaCLCa2 bearing both glutamates inherent only to anion/H^+^-transporters could indicate NO_3_^–^ specificity for the protein. This is in line with the presence of proline, specific for nitrate, in the second position of the selective filter GxGxPE (Figure 1, Table 1 and Table S1). To gain additional insight into function of SaCLCa2, site-directed mutagenesis of the nucleotide sequence of the gene encoding this protein was performed. A single nucleotide substitution C544T was introduced into the *SaCLCa2* sequence, leading to the replacement of proline, P182, by serine in the selectivity filter. Further complementation of growth defect of *Δgef1* by expression of this mutant *S. altissima* gene in *Δgef1* yeast cells was examined. When *Δgef1* strain was transformed with the construct *pMB1–SaCLCa2*(C544T), a partial growth restoration of yeast cells on YPD media containing Mn^2+^ occurred (Figure 3b), indicating likely the partial recovery of Cl^−^ transporter function.

Expression of SaCLCc2 with serine in the selectivity filter in yeast knockout mutant cells resulted in partial restoration of the mutant growth defect (Figure 3a), testifying in favor of chloride specificity of this protein.

**Figure 4:**
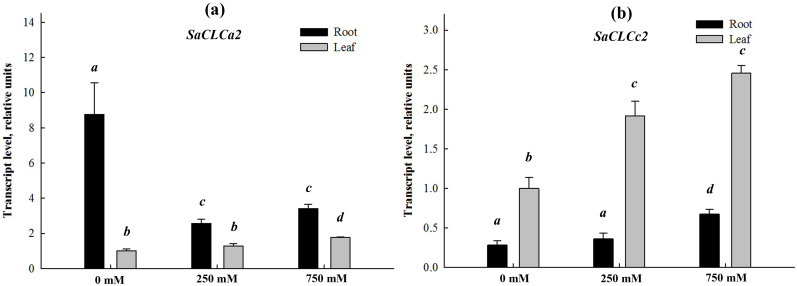
Relative abundance of *SaCLCa2* (**a**) and *SaCLCc2* (**b**) transcripts in roots (dark bars) and leaves (light bars) of *Suaeda altissima* plants grown at various NaCl concentrations in the plant growth medium. Data shown are means ± SE from three independent experiments. Each of them was performed in three analytical replicates. Bars with different letters are significantly different at *p* < 0.05.

To gain an insight into the physiological roles of SaCLCa2 and SaCLCc2 proteins, the effect of salinity on the expression of genes encoding these proteins in *S. altissima* organs was studied. The relative abundance of *SaCLCa2* and *SaCLCc2* transcripts were measured in roots and leaves of 45-day old plants grown on nutrient solution (NS) of Robinson and Downton (1985) [47] without NaCl or containing additionally NaCl in concentrations of 250 or 750 mM (Figure 4a,b). Plants grown without NaCl were characterized by a relatively high level of *SaCLCa2* transcripts in roots and the level was approximately an order of magnitude lower in leaves. The presence of NaCl led to a substantial decrease of *SaCLCa2* transcript abundance in roots and to a slight increase of that in leaves. In the absence of NaCl, the level of *SaCLCc2* expression in roots was more than an order lower in comparison with that of *SaCLCa2* in this organ, whereas in leaves, under control conditions, expression levels of the two genes were comparable. Under salinity, transcript abundance of *SaCLCc2* increased in both organs, but in roots remained at considerably lower levels than in leaves.

**Figure 5:**
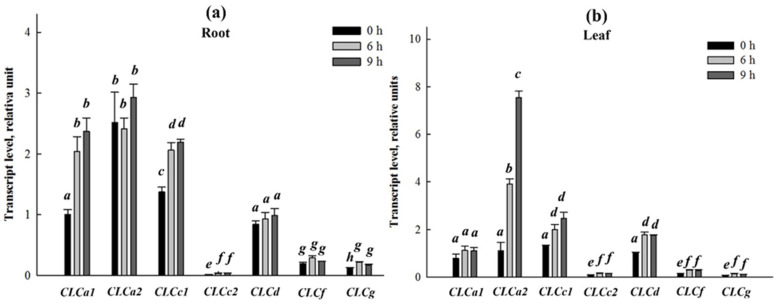
Relative transcript abundances of chloride channel family genes in roots (**a**) and leaves (**b**) of *Suaeda altissima* plants over the time course of salt stress. The plants were grown on the medium without NaCl followed by, at zero time-point, addition of NaCl at the final concentration of 250 mM. Data shown are means ± SE from three independent experiments. Each of them was performed in three analytical replicates. Bars with different letters are significantly different at *p* < 0.05.

The expression of *SaCLCa2* and *SaCLCc2* as well of other genes of the *S. altissima CLC* family [31,32,33] over time was also studied, at 0, 6, and 9 h after NaCl addition in the final concentration of 250 mM to NS of plants grown in the absence of NaCl (Figure 5a,b). In general, *SaCLCa1*, *SaCLCa2*, *SaCLCc1*, and *SaCLCd* showed relatively high expression activities in both roots and leaves. *SaCLCf* and *SaCLCg* displayed lower expression activities in these organs. The lowest expression activity was observed for *SaCLCc2*. Expression of *S. altissima CLC* genes demonstrated the ability to be induced by salt shock. In 6 and 9 h after addition of NaCl to NS, expression of *SaCLCa2*, *SaCLCc1*, *SaCLCd*, and *SaCLCf* increased in leaves and the expression of *SaCLCa1*, *SaCLCc1*, and *SaCLCg* increased in roots.

**Figure 6:**
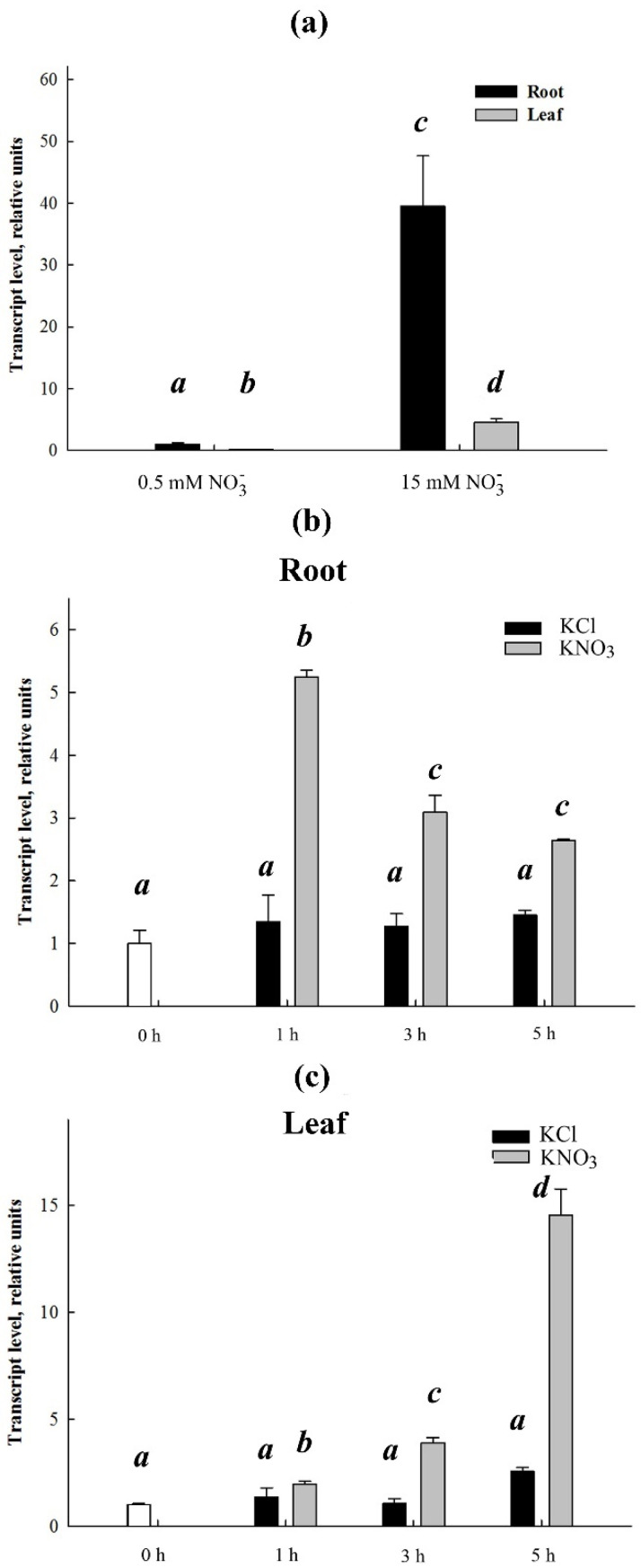
Effect of nitrate in the growth medium on expression of *SaCLCa2* in *Suaeda altissima*. (**a**) Relative transcript abundances of *SaCLCa2* in roots (dark bars) and leaves (light bars) of plants grown at nitrate deficiency (0.5 mM) or sufficient supply (15 mM) of nitrate under long-term experiments. Fast dynamics of relative abundance of *SaCLCa2* transcripts in roots (**b**) and leaves (**c**) of plants grown on nitrate deficiency (0.5 mM) medium after addition to the medium of KNO_3_ (light bars) or, as a control, KCl (dark bars) up to the final concentration of 5 mM. Transcript abundance levels are taken as 1 at zero time-points (white bars) when 5 mM of KCl or KNO_3_ was added. Data shown are means ± SE from three independent experiments. Each of them was performed in three analytical replicates. Bars with different letters are significantly different at *p* < 0.05.

The impact of NO_3_^–^ availability on transcript abundance of *SaCLCa2* (Figure 6), the gene characterized by high expression activity, was also studied. The 45-day old *S. altissima* plants were grown on the nutrient NS of Robinson and Downton (1985) [47] with the standard NO_3_^–^ concentration of 15 mM, or on the same NS but with the NO_3_^–^ concentration lowered to 0.5 mM by replacing a relevant portion of nitrate with chloride. The *SaCLCa2* expression in roots and leaves was found to be considerably lower in plants grown under nitrate deficit than at a sufficient supply of nitrate (40 and 50 times, respectively) (Figure 6a). Addition of KNO_3_ at the final concentration of 5 mM to the NS of plants grown under nitrate deficit (0.5 mM) resulted in considerable expression induction of *SaCLCa2* in both organs with time maxima in 1 h in roots (Figure 6b) and 5 h in leaves (Figure 6c). Addition of 5 mM KCl instead of 5 mM KNO_3_ to the NS did not induce the expression of *SaCLCa2*.

## 3. Discussion

In the current work, cDNAs of two members of the *CLC* family chloride channels, *SaCLCa2* and *SaCLCc2*, from euhalophyte *S. altissima*, have been cloned in addition to *SaCLCa1*, *SaCLCc1*, *SaCLCd*, *SaCLCf*, and *SaCLCg*, cloned previously [31,32,33]. Coding sequences of *SaCLCa2* and most parts of *SaCLCc2* are similar to the CDSs of *SfCLCa2* and *SfCLCc2*, respectively, identified earlier in silico in the de novo assembled transcriptome of *S. fruticosa*, a closely related species to *S. altissima* [31,35]. However, the nucleotide sequences of *SaCLCc2* cDNA at the 5´-end and amino acid sequence at the N-end of the encoded protein, respectively, are longer than those in the *S. fruticosa* homolog. The N-end of the SaCLCc2 amino acid sequence comprises additionally, in comparison with SfCLCc2, amino acids in positions 8–13, 16–18, and 36–124 (Figure S1).

The findings obtained here and in our previous works [31,32,33] show that amino acid sequences of identified *S. altissima* CLCs contain all key conserved motifs (Table 1) inherent in CLC proteins of other plants [29], mammals [26], and yeasts [48]. SaCLCa2 and SaCLCc2 proteins, as well SaCLCa1, SaCLCc1, and SaCLCd identified earlier (Figure 1, Table 1) [32,33] contain also both key conservative glutamates E1 and E2. These glutamates play a key role in functioning of anion/H+-antiporters of the CLC family [18]. E1 (gating glutamate) performs gating of the path for anions, whereas E2 (proton glutamate) is necessary for H^+^ transport and is present only in anion/H^+^ antiporters but not in anion channels of the CLC family [49]. The presence of both gating a glutamate and proton one in SaCLCa2 and SaCLCc2, as well in SaCLCa1, SaCLCc1, and SaCLCd suggests that these proteins are anion/H^+^ antiporters. Only gating, and only proton glutamates, were found in SaCLCf and SaCLCg, respectively, [33] (Table 1) suggesting that both these proteins are anion channels. The presence of serine in the selective filers [33] (Table 1) may indicate chloride selectivity of these proteins.

Sorting of the membrane proteins in plant cells, by analogy with mammals, was shown to be governed by cytosolic motifs that are recognized by adaptor proteins. These recruit components of the transport machinery, ensuring delivery of proteins to proper membranes [45,50,51]. Involvement of tyrosine- and dileucine-based motifs in sorting and control of subcellular localization of endocytosed plant proteins, such as auxin transporter PIN1 [40], vacuolar sorting receptor VSR4 [41], brassinosteroid receptor BRI1 [44], vacuolar transporters NRAMP3 и NRAMP4 [42], and SRK kinase [43], has been demonstrated. Analyses of amino acid sequences in *S. altissima* CLC proteins displayed presence of similar consensus motifs in N- and C-terminal domains located to cytoplasm (Figure 1, underlined).

Sorting signals present in protein cytosolic domains of eukaryotes include tyrosine-based motifs, YXXΦ, and dileucine-based motifs, DXXLL or [DE]XXXL[LI] [45,50,51]. Tyrosine containing motifs similar to YXXΦ are found in N- and C-termini of all identified SaCLCs. At the N-terminus of SaCLCg and at the C-terminus of SaCLCf, dileucine containing sequences DEESLL and DDKNLL, respectively, are found along with tyrosine containing sequences YXXΦ. In amino acid sequences of SaCLC proteins, there are also dileucine clusters, but with amino acid surroundings different from those in the consensus dileucine motifs [D/E]XXXL[L/I] or DXXLL.

Like other CLC proteins [26,29], SaCLCa2 and SaCLCc2 as well as other SaCLC proteins contain the regulatory cystathionine beta synthase (CBS) domains, CBS1 and CBS2, in the hydrophilic region at the C-terminus (Figure 1 and Figure S2, and Table 1).

**Figure 7:**
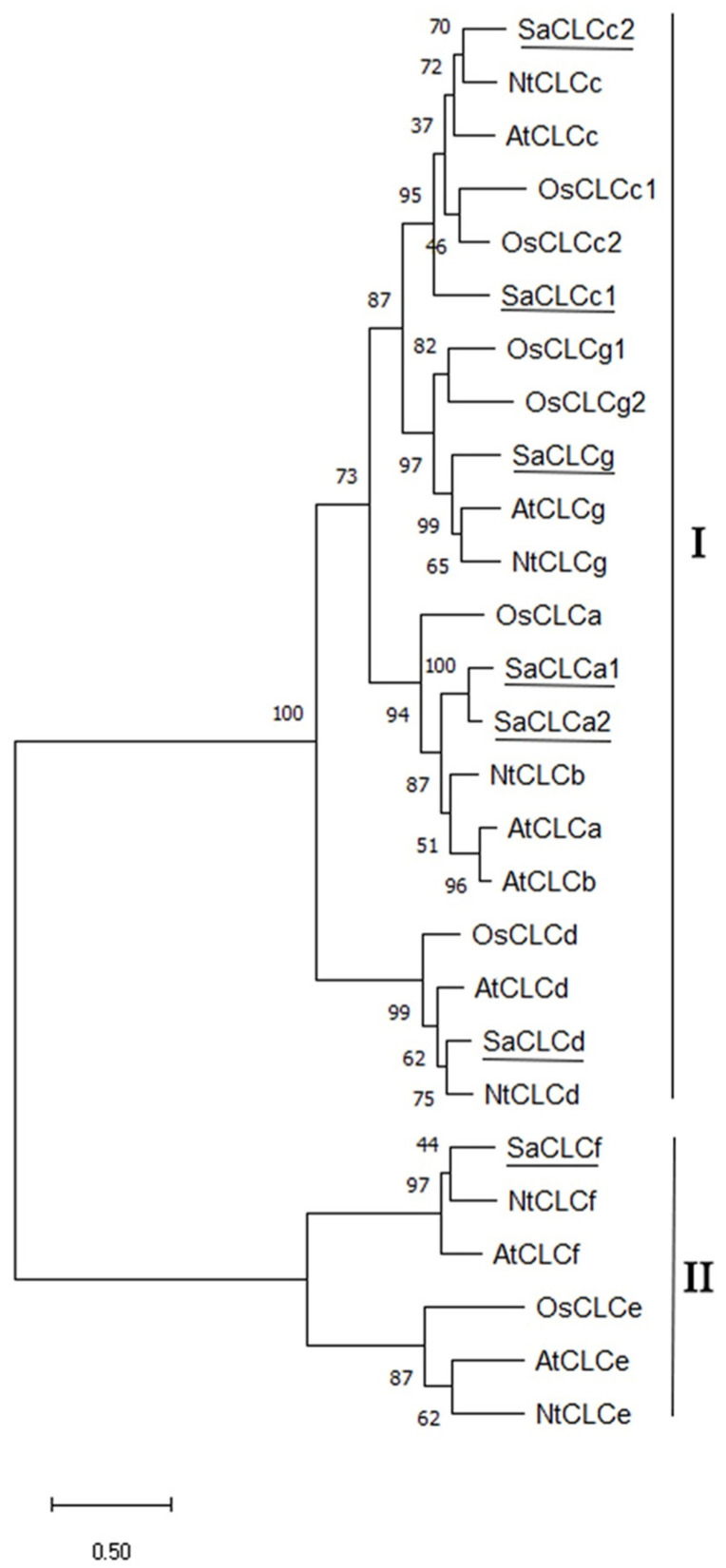
Phylogenetic tree of CLC family proteins of *Arabidopsis thaliana*, *Oryza sativa*, *Nicotiana tabacum*, and *Suaeda altissima*. All protein sequences were taken from the Protein Database (NCBI). Subgroup I is the “eukaryotic” branch, and subgroup II is the “prokaryotic” branch: AtCLCa (NP_198905.1), AtCLCb (NP_189353.1), AtCLCc (NP_199800.1), AtCLCd (NP_197996.1), AtCLCe (NP_567985.1), AtCLCf (NP_564698.1), AtCLCg (NP_198313.2), OsCLC1 (XP_015633162.1), OsCLC2 (XP_015622009.1), OsCLC3 (XP_015626588.1), OsCLC4 (AAO19370.1), OsCLC5 (XP_015636607.1), OsCLC6 (XP_015650515.1), OsCLC7 (XP_015620662.1), NtCLCc (NP_001312418.1), NtCLCb (NP_001312163.1), NtCLCd (XP_016512457.1), NtCLCe (XP_016461326.1), NtCLCf (XP_009787963.1), NtCLCg (XP_016468444.1), SaCLCa1 (ANG09048.1), SaCLCa2 (GenBank, acc. no. OM994378), SaCLCc1 (AVQ93350.1), SaCLCc2 (GenBank, acc. no. OM994379), SaCLCd (OK626332), SaCLCf (OK626333), and SaCLCg (OK626334). The phylogenetic tree was built in the MEGA 11 using the maximum likelihood method based on the Jones–Taylor–Thornton model. The number of bootstrap replicates was 1000; the values of bootstrap support are indicated near the nodes. Scale: 0.5 substitutions per site.

The chloride channel family has previously been shown to be subdivided into two subfamilies, the “eukaryotic” one and the “prokaryotic” one [19,52]. The first subfamily is characterized by higher homology with eukaryotic (human) CLCs, while representatives of the second subfamily are closer to prokaryotic CLCs [19,52]. On the CLC cladogram obtained in this study, plant proteins are also divided into two clusters (Figure 7). The first cluster (I) includes proteins homologous to AtCLCa–d and AtCLCg, the CLC proteins which are of eukaryotic origin, and the second one (II) includes proteins homologous to AtCLCe and AtCLCf, which are assumed to be of prokaryotic origin. SaCLCa2 and SaCLCc2, as well as the previously identified SaCLCa1, SaCLCc1, SaCLCd, SaCLCg [31,32,33], belong to the “eukaryotic” subfamily, while the SaCLCf protein relates to the “prokaryotic” one.

The ionic specificity of SaCLCa2 and SaCLCc2 was investigated by heterologous expression of these proteins in the *S. cerevisiae* mutant strain *Δgef1*, in which the single CLC gene, GEF1, was disrupted [48]. The product of this gene, a chloride transporter Gef1p, supposedly a Cl^−^/H^+^ antiporter, localized to Golgi, vacuolar and plasma membranes in yeast cells [34]. Multiple defects in cell processes contributing to the complex phenotype of *Δgef1* strains were outlined previously [34,46]. One of the mutant strain phenotype manifestations was inability of the mutant cells to grow on rich YPD medium containing toxic cations including Mn^2+^ [46]. The authors of this study hypothesized, that Cl^−^/H^+^ exchange was required for stimulating vacuolar H^+^-ATPase, and probably vacuolar H^+^-translocating inorganic pyrophosphatase (H^+^-PPase), which in turn was necessary for ΔpH-dependent compartmentalization of Mn^2+^ ions in vacuoles. The growth of the *Δgef1* strain obtained by us was also inhibited on YPD medium supplemented with Mn^2+^ ions and a partial growth restoration was observed under some conditions. The following data obtained in our experiments argue in favor of *SaCLCa2* nitrate specificity: (1) Expression of *SaCLCa2* in cells of the mutant did not result in complementation of the Mn^2+^-mediated growth defect (Figure 3a) arising in the mutant due to absence of the Cl^−^ transporter Gef1p. (2) Partial growth restoration of *Δgef1* on Mn^2+^ containing medium was observed when the mutant was transformed with *SaCLCa2(C544T)* gene (Figure 3b), the product of which contained serine (specific for chloride) instead of proline (specific for nitrate) in the selective filter. (3) Stimulation of *SaCLCa2* expression by KNO_3_, but not KCl addition to the NS occurred in *S. altissima* organs (Figure 6a–c). (4) The proline residue specific for nitrate was found in the selective filter of the *SaCLCa2* gene (Figure 1; Table 1).

Stimulation of *SaCLCa2* expression, observed in leaves in response to 250 mM NaCl addition to NS (Figure 5b) may be attributed to necessity of nitrate redistribution among organs and tissues under conditions of nitrate competition with chloride for anion transporters under salinity. Such competition was suggested in a number of studies [53,54,55,56].

The results obtained indicate that another gene cloned from *S. altissima* in the current work, *SaCLCc2*, characterized in general by low expression in both roots and leaves, most likely encodes a chloride transporter. The following observations support this assumption: (1) partial rescue of the Mn^2+^-mediated growth defect of the *Δgef1* mutant when transformed by *SaCLCc2* (Figure 3a); (2) a stimulation of *SaCLCc2* expression in roots and leaves of *S. altissima* by NaCl under conditions of both constant long-term salinity (Figure 4b) and in response to salt shock (Figure 5a,b): (3) the presence of a serine residue specific for chloride in the selective filter of *SaCLCc2* encoded protein (Figure 1, Figure S2, and Table 1).

Taking into account the data obtained earlier [31,32,33], altogether 7 representatives of CLC family in the halophyte *S. altissima* (Table 1)—which corresponds to 7 full size or partial CDSs found in silico in transcriptomes of a close related species *S. fruticosa* [31,35]—are identified by us. Two of the identified proteins, SaCLCa1 and SaCLCa2, are likely to be NO_3_^−^/H^+^ antiporters, while the other proteins are likely to be Cl^−^/H^+^ antiporters (SaCLCc1, SaCLCc2, SaCLCd) or chloride channels (SaCLCf, SaCLCg).

The list of identified CLC proteins in *S. altissima* is probably not full, because the presence of *CLC* genes expressed at very low levels, in a tissue-specific manner or under specific environmental conditions is not excluded. A full set of the *S. altissima* CLC genes is expected to be identified as a result of genome sequencing of this species. The presence of CLC isoforms, besides those cloned, which occur via alternative splicing is also possible. Longer isoforms of SaCLCf and SaCLCc2 as well shorter isoforms of SaCLCa1, SaCLCa2, SaCLCc1, SaCLCg and SaCLCd may exist in *S. altissima*. Variation of 3’- or 5’- end cDNA sequences could be a regulatory mechanism of CLC localization and, accordingly, anion fluxes and anion compartmentalization in plant cells.

Conserved dileucine clusters and tyrosine residues found in N-termini of SaCLCs (Figure 1) suggest the existence of signal sequences defining subcellular localization of CLS proteins in *S. altissima* cells. Particularly, the presence of dileucine clusters at N-termini of SaCLCa1, SaCLCa2, SaCLCc1, SaCLCg, and SaCLCf may define localization of these proteins to intracellular compartments. In mammals, dileucine motifs are shown to be involved in interaction of CLCs with clathrin, internalization of the CLCs, and targeting the CLCs to intracellular membranes [51,57]. The absence of dileucine motifs in SaCLCc2 and SaCLCd N-termini may result in preferential targeting of these proteins to surface membranes.

Stimulation of *SaCLCc1*, *SaCLCc2*, *SaCLCd*, *SaCLCf*, and *SaCLCg* expression in the *S. altissima* organs in response to NaCl addition to the growth medium has been demonstrated in the current work (Figure 4 and Figure 5) and previously [31,32,33]. These findings may indicate involvement of the proteins encoded by these genes in Cl^−^ transport and maintenance of Cl^−^ homeostasis when *S. altissima* plants are subjected to NaCl stress. There is no doubt about the important roles of anion/H^+^ antiporters under salinity in Cl^−^ compartmentalization in vacuoles and pH regulation in lumen of cytoplasmic organelles, as well in nitrate storage in vacuoles and regulation of cytosol concentrations of nitrate. The *Arabidopsis* homolog AtCLCa has been shown to participate both in the accumulation of anions in the vacuole during stomatal opening and anion release into the cytosol during stomatal closure in response to the stress hormone abscisic acid [27,28].

**Figure 8:**
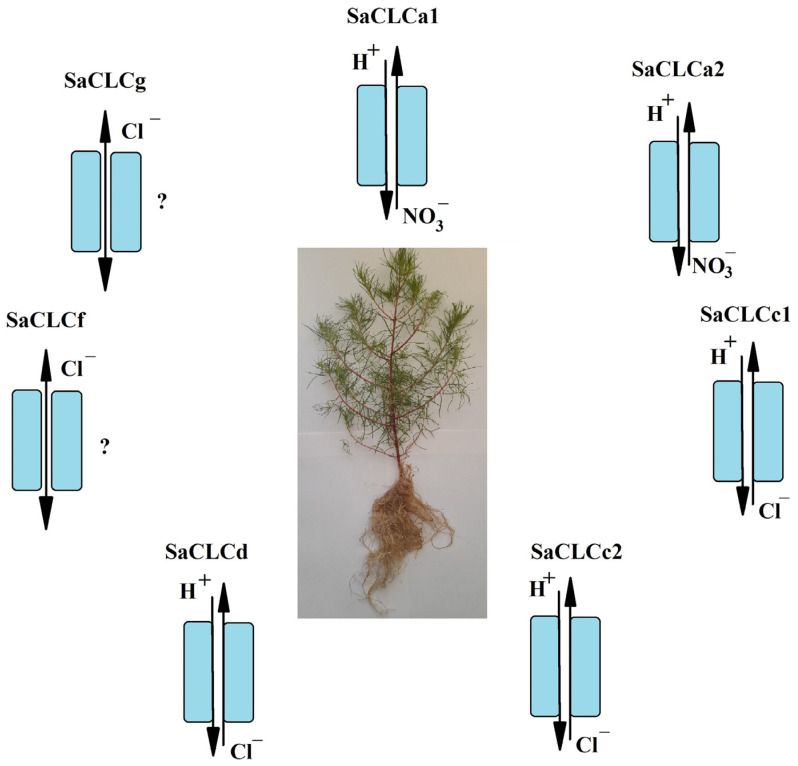
CLC genes/transporters family in *Suaeda altissima*.

Increase in expression of the two genes presumably encoding NO_3_^−^/H^+^ antiporters, *SaCLCa1* and *SaCLCa2*, in response to NaCl addition to the growth medium (Figure 5) may be caused by nitrate deficit arising due to competition between NO_3_^−^ and Cl^−^ ions for anion transporters and channels [53,54,55,56]. These findings may also imply that physiological roles of the proteins encoded by these genes are organ specific because expression stimulation in response to NaCl addition in *SaCLCa1* occurred in roots (Figure 5a), whereas in *SaCLCa2* did in leaves (Figure 5b). It is conceivable that all CLC genes identified in euhalophyte *S. altissima* could be implicated in mechanisms underlying salt tolerance (Figure 8).

## 4. Materials and Methods

### 4.1. Plant Material

Seeds of *S. altissima* were germinated in wet sand at 24 °C. After three weeks, the seedlings were transplanted into 3-L glass containers (4 plants per container) on an aerated Robinson and Downton [47] nutrient solution. After 7 days of growth, NaCl was added to the Robinson-Downton NS at final concentrations of 0, 250, and 750 mM NaCl (Figure 4, Table S2). To avoid salt shock, NaCl addition to the NS was performed gradually with increment of 50, or 100 mM. Salt stress (Figure 5) was carried out simultaneously by adding up to 250 mM NaCl to the nutrient medium (150 mL 5 M NaCl per 3-L container). Growth of *S. altissima* plants proceeded in a growth chamber under controlled environmental conditions in water culture at 24 °C and air relative humidity of 60–70%. The plants were illuminated with high pressure sodium lamps DNaZ_400 (Reflux, Russia) at photoperiod 16 h/8 h (day/night) and a light intensity of 300 μmol photons/(m^2^ · s). Forty-five days old plants were used in the experiments (Figure S3).

For gene expression studies by qRT-PCR, plants were grown on Robinson and Downton (1985) NS [47] at a standard concentration of NO_3_^−^ (15 mM) with addition of NaCl (0 mM, 250 mM and 750 mM) or on the same media, but with a lowered NO_3_^−^ concentration to 0.5 mM (Table S2) and subsequent addition of nitrate or chloride to a final concentration of 5 mM. For total RNA extraction, leaves and roots of *S. altissima* were sampled and frozen in liquid nitrogen for future use.

### 4.2. Extraction of Total RNA from Plant Material and Synthesis of First Strand cDNA

Total RNA was isolated from roots and leaves of *S. altissima* by the hot phenolic method [58] and used as template for the first-strand cDNA synthesis. A 0.5 g sample was frozen in liquid nitrogen and ground into powder, then 1 mL of acid phenol (pH 4.5) with RNA extraction buffer (0.1 M LiCl, 0.1 M Tris-Cl pH7.5, 1% SDS, 10 mM EDTA) in a ratio 1:1 was added. Chloroform (0.5 mL) was added to the mixture and shaken on a vortex for 5 min (30 min at 20,000× *g*). Purification with chloroform was repeated 2 times (5 min, 20,000× *g*). 10 M LiCl was added to the supernatant to a final concentration of 2 M and incubated for 16–18 h at 4 ℃ (30 min, 12,000× *g*, 4 ℃). The RNA pellet was washed 2 times with 2 M LiCl and 1 time with 80% ethanol (5 min, 12,000× *g*, 4 ℃). The precipitate was dissolved in mQ water. RNA concentration was measured using NanoDrop ND1000 (Thermo Fisher Scientific, Inc., Waltham, MA, USA) by the ratio A_260_/A_280_. The quality of isolated RNA was also confirmed by electrophoresis in 1% agarose gel.

The first cDNA strand was obtained with reverse transcriptases MINT (for amplification of the 3’- and 5’-ends CLC transcript sequences using Step-Out RACE technology) or MMLV (for cloning full-length CDS coding sequences and for performing qRT-PCR) according to the manufacturer’s protocol (“Evrogen”, Moscow, Russia). 1 µg of total RNA was treated with DNase I, RNase-free (“Fermentas”, Thermo Fisher Scientific, Inc., Waltham, MA, USA, kat# EN0525), according to the manufacturer’s recommendations. 10 µL of the mixture (1 µg RNA, 1 µL 10×reaction buffer with MgCl_2_, 1 µL (1 U) DNase I, H_2_O to 10 µL) was incubated for 30 min at 37 ℃, then 1 µL of 50 mM EDTA was added and the mixture was incubated for 10 min at 65 ℃. For the synthesis of the first cDNA strand, 9 µL of a mixture containing 2 µL of H_2_O, 5 µL (0.5 µg) of RNA after treatment with DNase I, and 2 µL of 20 µM primer (dT)_15_, were heated for 2 min at 70 ℃. 11 µL of a mixture containing 2 µL H_2_O, 4 µL 5 × buffer, 2 µL 10 mM dNTPs, 2 µL 20 mM DTT, 1 µL MMLV reverse transcriptase was added and incubated first for 60 min at 42 ℃ and then for 10 min at 70 ℃. For subsequent reactions, 50–100 ng of the obtained cDNA per reaction was used.

For the amplification of 3’- and 5’-ends CLC transcript sequences using Step-Out RACE technology, synthesis of first-strand cDNA was executed with Mint reverse transcriptase (“Evrogen”, Moscow, Russia), according to the protocol of the manufacturer. For cloning cDNA of genes and quantitative analysis of *SaCLCa2* and *SaCLCc2* transcript levels in *S. altissima* organs, synthesis of first-strand cDNA was carried out using total RNA, (dT)15 primer and MMLV reverse transcriptase (“Evrogen”, Moscow, Russia).

### 4.3. Cloning of SaCLCa2 and SaCLCc2

Firstly, we performed an in silico search for the sequences homologous to the *AtCLCa*, *AtCLCb*, and *AtCLCc* genes in the de novo transcriptomes of *Suaeda fruticosa* (L.) Forssk, a closely related species of *S. altissima,* assembled by us earlier [31,35]. To do this, the contigs of the assembled transcriptomes were translated into amino acid sequences and, in the obtained arrays, the search for the sequences related to the CLC family proteins was accomplished. AtCLCa, AtCLCb and AtCLCc proteins were used as queries. Four contigs were found in the transcriptomes that included full-length coding sequences of the chloride channel family genes and named after their similarity to the corresponding *A. thaliana* CLCs: *SfCLCa1*, *SfCLCa2*, *SfCLCc1*, and *SfCLCc2*. The coding sequences of the *SaCLCa1* (KX013489.1) and *SaCLCc1* (MG670589.1) genes of *S. altissima* corresponding to the *SfCLCa1* and *SfCLCc1* contigs of *S. fruticosa*, were cloned by us earlier [31,32]. To the contigs identified in *S. fruticosa* transcriptomes, *SfCLCa2* and *SfCLCc2*, the primer pairs SaCLCa2R and SaCLCa2F, SaCLCc2F and SaCLCc2R, respectively, were designed whereby the partial coding sequences of *SaCLCa2* and *SaCLCc2* CDS were obtained. The partial cDNA fragments were amplified from cDNA template using Encyclo DNA polymerase (“Evrogen”, Moscow, Russia) and sequenced.

Based on the partial *SaCLCa2* and *SaCLCc2* sequences obtained, the forward primer sets (1) SaCLCa2_3’RACE_F1 (first PCR round), SaCLCa2F (second PCR round), (2) SaCLCc2F (first PCR round), SaClCc2_F1 (second PCR round) for amplification of 3’-end fragments and the reverse primer sets (1) SaCLCa2R (first PCR round), SaCLCa2_5’RACE_R1 (second PCR round), SaCLCa2_5’RACE_R2 (third round PCR), (2) SaCLCc2R (first PCR round), SaCLCc2_5’RACE_R1 (second PCR round) for amplification of 5’-end fragments *SaCLCa2* and *SaCLCc2* were designed (Table S3). With these primers, we amplified the 3’- and 5’-end fragments of SaCLCa2 and SaCLCc2 (~1000–1500 bp) by 3’- and 5’-rapid amplification of cDNA ends (3’- and 5’-RACE) using the Step-Out RACE technology, cloned them into vector pAL2-T (“Evrogen”, Moscow, Russia) and sequenced. Partial sequences (central fragments, 3’- and 5’-end fragments of *SaCLCa2* and *SaCLCc2*) were combined in silico and the resulting complete coding sequences *SaCLCa2* and *SaCLCc2* contained open reading frames (ORFs) for proteins of 800 and 708 amino acids (aa), respectively. Full-length *SaCLCa2* and *SaCLCc2* cDNAs were amplified with a CloneAmpPCR PreMix kit (Clontech, Mountain View, CA, USA) using the pairs of primers: pVR2_SaCLCa2_F and pVR2_SaCLCa2_R, pVR2_SaCLCc2_F and pVR2_SaCLCc2_R (Table S3), and total first strand cDNA as a template. The amplified *SaCLCa2* and *SaCLCc2* cDNAs fragments were cloned into yeast vector pVR2 [59] using Gibson Assembly Cloning Kit (NEB, USA), and sequenced. For expression in the yeast mutant *Δgef1*, the full-length coding sequences *SaCLCa2* and *SaCLCc2* were amplified with primer pairs pMB1_SaCLCa2_F and pMB1_SaCLCa2_R, pMB1_SaCLCc2_F and pMB1_SaCLCc2_R (Table S3), using the pVR2–*SaCLCa2* and pVR2–*SaCLCc2* constructs, respectively, as templates, and cloned in yeast expression vector pMB1 under control of a strong constitutive promoter *GPD1* [60]. A linear form of pMB1 was amplified using pair of primers, pMB1_F and pMB1_R (Table S3). For amplification of SaCLCa2 and SaCLCc2 coding sequences, total RNA was isolated from roots and leaves, respectively. The cloned coding sequences of *SaCLCa2* and *SaCLCc2* genes were sequenced and the sequences obtained were deposited in GenBank.

### 4.4. Heterologous Expression of the SaCLCa2 and SaCLCc2 Genes in Δgef1 Yeast Mutant

*S. cerevisiae* mutant strain *Δgef1* obtained by us earlier from the wild type strain W3031A (MATa *leu2*, *his3*, *trp1*, *ura3*, *ade2*) [31,32] was transformed with constructs pMB1– *SaCLCa2* and pMB1–*SaCLCc2* by the lithium protocol [61]. To explore the growth characteristics of the mutant strain *Δgef1* and the transformants, yeast cells were plated on an agarised (2%) selective rich YPD medium consisting of 1% yeast extract, 2% peptone, 2% dextrose (as a fermentable carbon source). To study the effect of Mn^2+^ on yeast cell growth MnCl_2_/MnSO_4_ at final concentrations of 2 or 3 mM was added to the medium.

### 4.5. Site-Directed Mutagenesis of SaCLCa2(C544T)

A single nucleotide substitution in the *SaCLCa2* sequence, C544T, was introduced by inverse PCR on pMB-*SaCLCa2* using a pair of primers, SaCLCa2_P182S_F and SaCLCa2_528_R (Table S3), and a CloneAmp Hi-Fi PCR premix kit (Clontech, Takara, Saint-Germain-en-Laye, France). The resulting linear form of pMB-*SaCLCa2 (C544T)* was converted to the circular form with T4 DNA ligase (“Sibenzyme”, Novosibirsk, Russia) and propagated in *E. coli* cells. The substitution was validated by nucleotide sequencing of the resulting recombinant plasmid.

### 4.6. Quantitative Analysis of SaCLCa2 and SaCLCc2 Transcripts in S. altissima Organs

The cDNA templates for *SaCLCa2* and *SaCLCc2* fragment amplification were synthesized on the templates of total RNAs isolated from roots and leaves of *S. altissima* plants grown on nutrient media with various NaCl concentrations or subjected and not subjected to NaCl shock. Quantitative analysis of *SaCLCa2* and *SaCLCc2* transcripts was performed by qRT-PCR method using a LightCycler^®^ 96 System (“Roche Diagnostics Corporation”, Indianapolis, IN, USA). A reaction mixture with intercalating dye SYBR Green I (Evrogen, Russia) was used. To amplify the *SaCLCa2* and *SaCLCc2* fragments, pairs of primers, the sequences of which are given in Table S3, were used. Target gene mRNA expression levels were normalized for *S. altissima* actin gene, *SaAct7* (GenBank, acc. no. MK615596.1) and factor elongation 1 alpha gene, *SaeEF1alpha* (GenBank, acc. no. MN076325.1). To amplify the *SaAct7* and *SaeEF1alpha* fragments, primer pairs, SaAct_F1 and SaAct_R1, SaeEF1alfa_F1 and SaeEF1alfa_R1, respectively, were used (Table S3). Results are based on three replicates. The results obtained were processed by the LightCycler 96SW 1.1 software. Similar results were obtained with *SaAct7* and *SaeEF1alpha;* hence the data are presented only with the former gene.

### 4.7. Primer Design

Primers for qRT-PCR- experiments were designed by Light Cycler96 Probe Design software (version 2.0, https://www.oligo.net/, accessed on 10 December 2022). Other primers were designed using Oligo 7 software (version 7, https://www.oligo.net/, accessed on 10 December 2022) or Primer-Blast software (https://www.ncbi.nlm.nih.gov/tools/primer-blast/, accessed on 10 December 2022). All primers used in the study are listed in Table S3.

### 4.8. Bioinformatic Analysis of Amino Acid Sequences

Alignment of amino acid sequences was performed by MAFFT software (https://www.ebi.ac.uk/Tools/msa/mafft/, accessed on 7 December 2022). A phylogenetic tree of plant CLC family proteins was created by Molecular Evolutionary Genetic Analysis (MEGA) 11 software (version 11, https://www.megasoftware.net/, accessed on 7 December 2022), using the maximum likelihood method based on the Jones–Taylor–Thornton model [62] (1000 bootstrap replication performed). Protein topology was predicted by Philius (version 3.0, https://noble.gs.washington.edu/proj/philius/, accessed on 15 December 2022) software.

### 4.9. Statistical Analysis

Data processing (mean, standard errors) and graphs were performed using Sigma Plot software (version 14.0). A Shapiro–Wilk normality test followed by a Welch’s *t* test was applied. Statistical calculations were carried out in the Microsoft Excel program (version 2019). Standard errors are given. Different letters indicate significant difference (*p*-value < 0.05).

## Figures and Tables

**Table 1:** Features of chloride channel family proteins from the euhalophyte *Suaeda altissima* (calculated parameters).

	SaCLCa1	SaCLCa2	SaCLCc1	SaCLCc2	SaCLCd	SaCLCf	SaCLCg
mRNA, complete cds (GenBank ID)	KX013489.1	OM994378	MG670589.1	OM994379	OK626332.1	OK626333.1	OK626334.1
Number of amino acids	811	800	791	708	793	587	776
Subunit size (kDa)	89.5	88.2	87.0	77.5	87.6	62.3	85.8
pI *	8.55 ± 0.09	8.48 ± 0.07	7.91 ± 0.35	7.91 ± 0.11	8.15 ± 0.12	6.62 ± 0.33	8.07 ± 0.13
GRAVY index *	0.209	0.272	0.314	0.385	0.202	0.249	0.395
Subcellular localization **	vacuole (*p* = 0.82 ± 0.1)	vacuole (*p* = 0.77 ± 0.0)	vacuole (*p* = 0.82 ± 0.1)	vacuole (*p* = 0.75 ± 0.1)	vacuole (*p* = 0.67 ± 0.1)	vacuole/mitochondrion (*p* = 0.34 ± 0.08/ *p* = 0.17 ± 0.02)	vacuole (*p* = 0.64 ± 0.07)
The CLC proteins of *A. thaliana* with highest homology scores	AtCLCb (75.33% Identity)	AtCLCb (77.23% Identity)	AtCLCc (69.60% Identity)	AtCLCc (75.04% Identity)	AtCLCd (79.75% Identity)	AtCLCf (70.44% Identity)	AtCLCg (71.09% Identity)
Function	NO_3_^–^/H^+^-exchange	NO_3_^–^/H^+^- exchange	Cl^−^/H^+^- exchange	Cl^−^/H^+^- exchange?	Cl^−^/H^+^- exchange	Cl^−^-channel?	Cl^−^-channel?
Selectivity filter	GPGIP	GPGIP	GSGIP	GSGIP	GSGIP	SSKSSQ	GSGIP
E_148_ (Eg) ***	E	E	E	E	E	E	A
E_203_ (Ep) ***	E	E	E	E	E	T	E

* pI and GRAVY index are calculated using several software tools (https://web.expasy.org/compute_pi/, accessed on 10 December 2022; https://www.ebi.ac.uk/Tools/seqstats/emboss_pepstats/, accessed on 10 December 2022; https://www.bioinformatics.org/sms2/protein_iep.html, accessed on 10 December 2022) and GRAVY calculator (version 27.11.2011, http://www.gravy-calculator.de/, accessed on 12 December 2022), respectively. pIs shown are mean ± SD from three programs. ** Subcellular localization of proteins was predicted using Microsoft DeepLoc-1.0: Eukaryotic protein subcellular localization predictor (http://www.cbs.dtu.dk/services/DeepLoc-1.0/index.php, accessed on 16 December 2022), ProtComp (http://linux1.softberry.com/berry.phtml?topic=index&group=programs&subgroup=proloc, accessed on 16 December 2022), and Protein Prowler (http://bioinf.scmb.uq.edu.au:8080/pprowler_webapp_1-2/index.jsp, accessed on 16 December 2022). *P*_sub.loc_ shown are mean ± SD from three programs. *** Gating (E148) and proton (E203) glutamates EcCLC (CLC from *E.coli*), respectively.

## Data Availability

All data included in this study are available upon request by contact with the corresponding authors. The cloned *SaCLCa2* and *SaCLCc2* cDNA were deposited in GenBank (acc. no. OM994378 and, OM994379, respectively).

## Supplementary Materials

The supporting information can be downloaded at: https://www.mdpi.com/article/10.3390/ijms24020941/s1.
