# Supplementary material for: Chloride Channel Family in the Euhalophyte Suaeda altissima (L.) Pall: Cloning of Novel Members SaCLCa2 and SaCLCc2, General Characterization of the Family
Source: Int J Mol Sci. 2023 Jan 4;24(2):941. doi: 10.3390/ijms24020941 (PMC9867446; doi:10.3390/ijms24020941)
Supplement: Supplementary file 1 [file ijms-24-00941-s001.zip › ijms-2074214-supplementary.pdf]

**Supplementary materials**

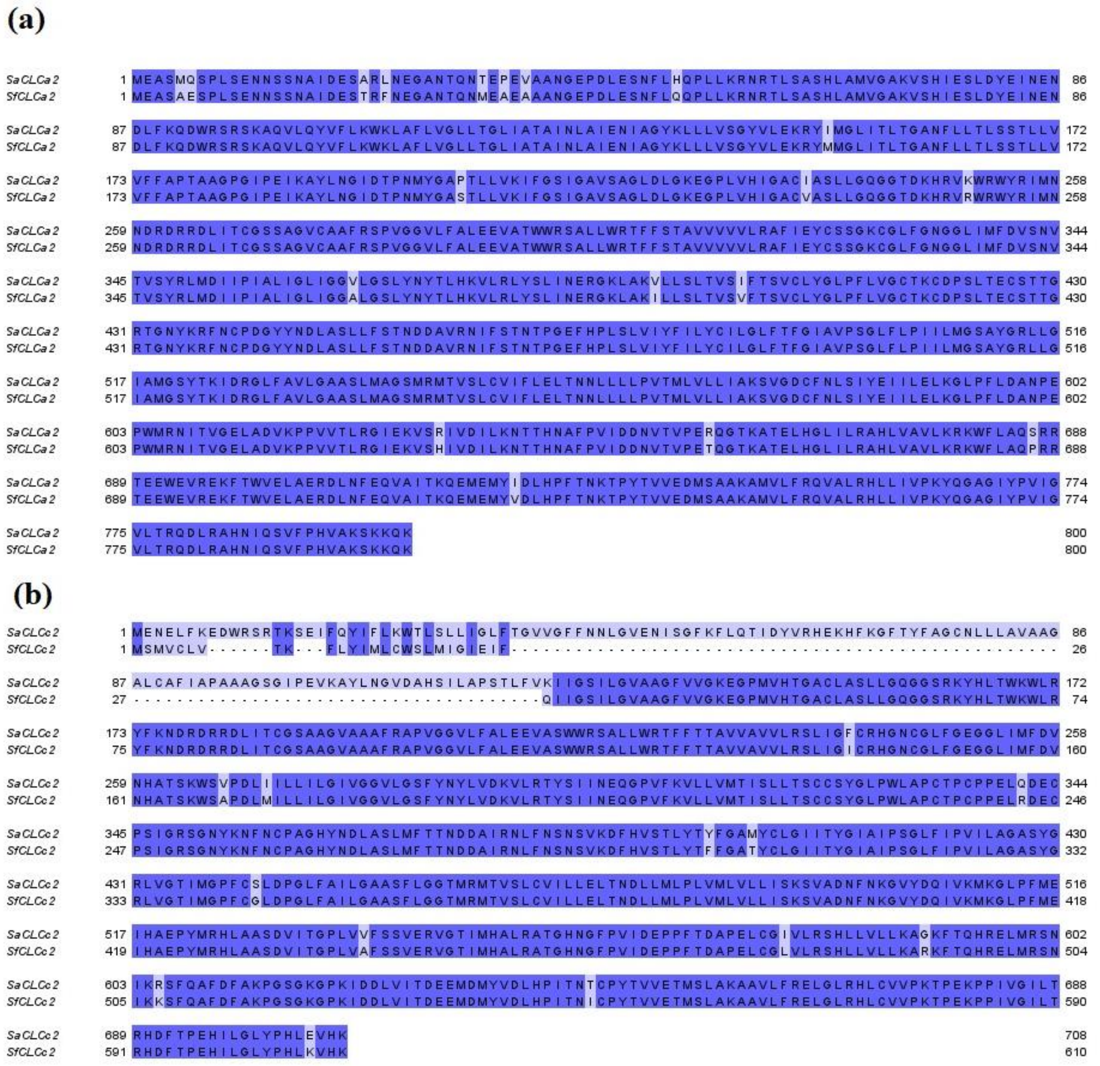

**Figure S1.** Alignment of the proteins from *Suaeda altissima*, SaCLCa2 (a) and SaCLCc2 (b), with their homologs from closely related species *Suaeda fruticosa*, SfCLCa2 (a) and SfCLCc2 (b), respectively. SaCLCc2, unlike to SaCLCa2, is shortened from N-terminus.

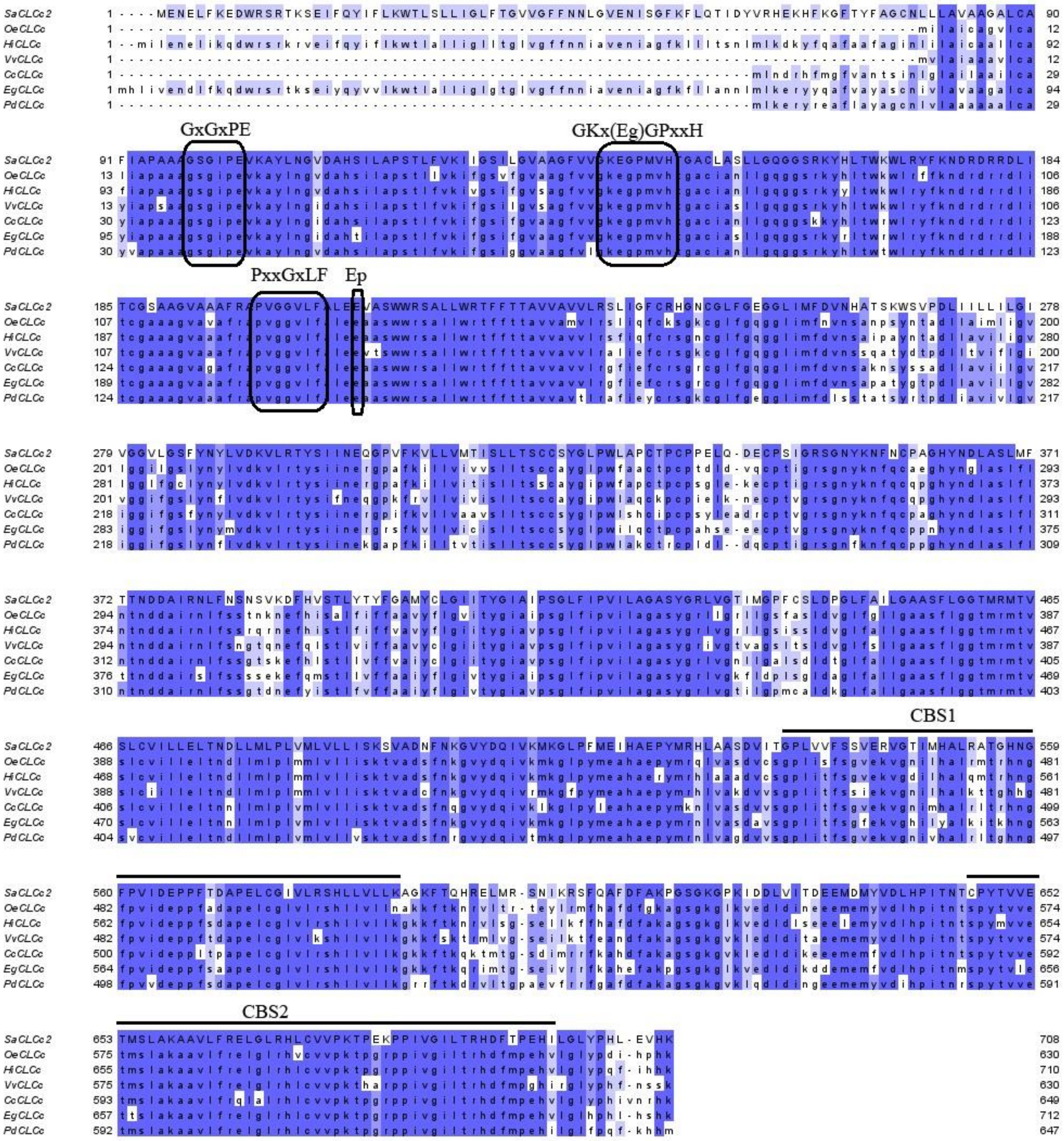


**Figure S2.** The CLCc proteins found in transcriptomes of different plants: *Suaeda altissima* (L.) Pall (GenBank, acc. no. OM994379), *Olea europaea* subsp. *europaea* (CAA 2963713.1), *Handroanthus impetiginosus* (PIN10391.1), *Vitis vinifera* (XP_010657886.1), *Olea europaea* var. *sylvestris* (XP_022861637.1), *Eucalyptus grandis* (XP 010030224.1), *Phoenix dactylifera* (XP_038975861.1).

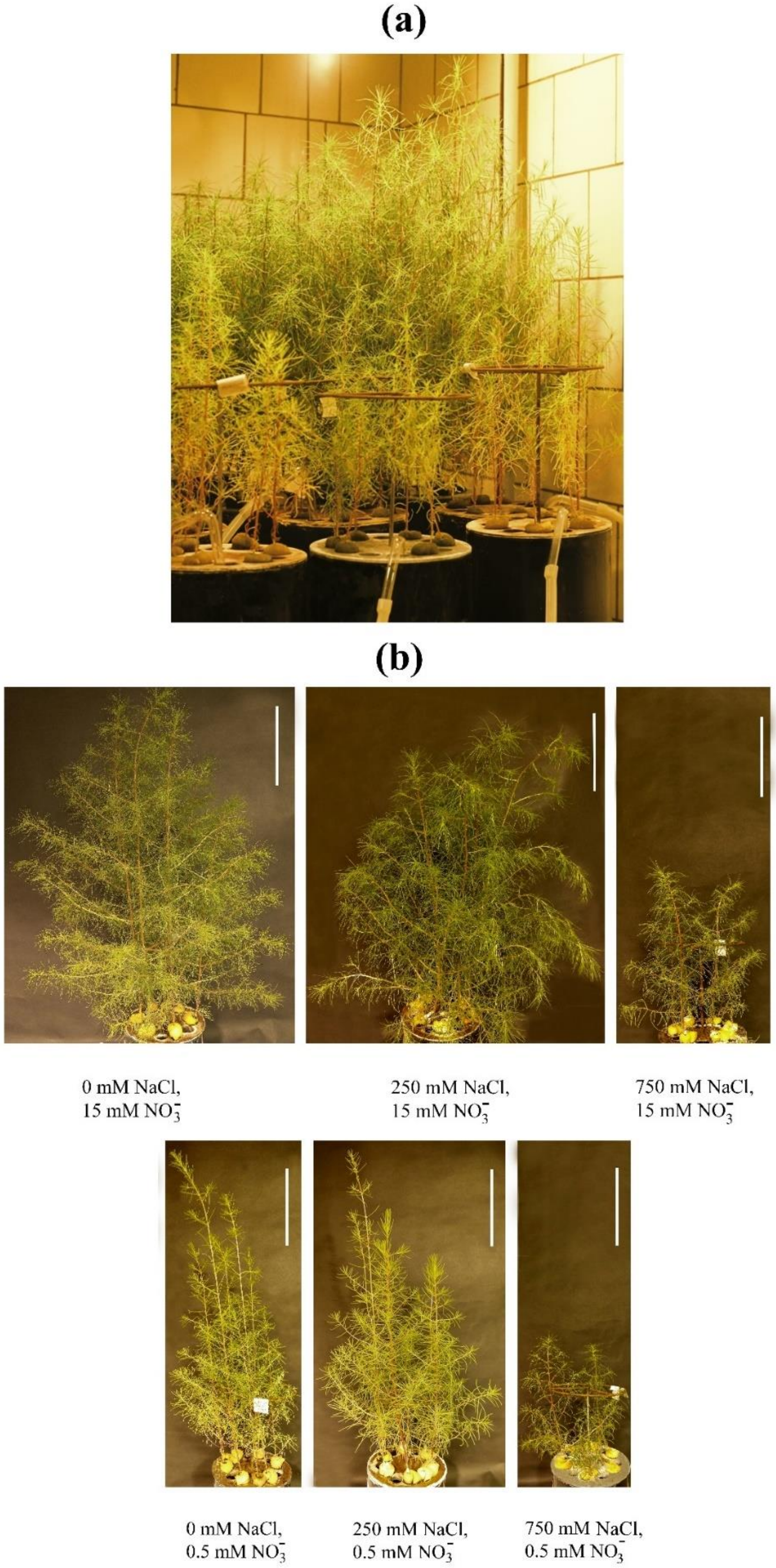


**Figure S3.** *Suaeda altissima* plants grown under different concentrations of NaCl and nitrate in the aerated nutrient medium. **(a)** General view of the plants in the growth chamber. **(b)** *S. altissima* 45-day-old plants grown under different concentrations of NaCl (0, 250, 750 mM) and nitrate (15 mM or 0.5 mM) in the nutrient medium. Scale bar represents 15 cm.

**Table S1.** Conserved amino acids motifs and residues and their positions in SaCLCa2 and SaCLCc2 sequences.

| Conserved amino acids motifs and residues | SaCLCa2 | SaCLCc2 |
|---|---|---|
| Gating Glu | E 225 | E 142 |
| Proton Glu | E 292 | E 209 |
| (1) residues in the selectivity filter, a.a. | G**P**GIPE 181–186 | G**S**GIPE 98–103 |
| (2) GKxGPxxH, a.a. | GKEGPLVH 223–230 | GKEGPMVH 140–147 |
| (3) PxxGxLF, a.a. | PVGGVLF 282–288 | PVGGVLF 199–205 |
| CBS1, a.a. | 619–677 | 535–587 |
| CBS2, a.a. | 733–786 | 646–697 |

**Table S2.** The composition of the nutrient media used in the research.

<table>
<tr><td>№</td><td colspan="2">15 mM $NO_3^-$</td><td>№</td><td colspan="2">0.5 mM $NO_3^-$</td></tr>
<tr><td>1.</td><td>0 mM NaCl</td><td rowspan="3">4 mM $Ca(NO_3)_2$<br>7 mM $KNO_3$<br>1 mM $KH_2PO_4$<br>2 mM $MgSO_4$<br>50 µM $FeSO_4$<br>50 µM $Na_2EDTA$<br>50 µM $H_3BO_3$<br>10 µM $MnCl_2$<br>1 µM $ZnSO_4$<br>0.5 µM $CuSO_4$<br>0.1 µM $Na_2MoO_4$</td><td>4.</td><td>0 mM NaCl</td><td rowspan="3">4 mM $CaCl_2$<br>6.5 mM KCl<br>0.5 mM $KNO_3$<br>1 mM $KH_2PO_4$<br>2 mM $MgSO_4$<br>50 µM $FeSO_4$<br>50 µM $Na_2EDTA$<br>50 µM $H_3BO_3$<br>10 µM $MnCl_2$<br>1 µM $ZnSO_4$<br>0.5 µM $CuSO_4$<br>0.1 µM $Na_2MoO_4$</td></tr>
<tr><td>2.</td><td>250 mM NaCl</td><td>5.</td><td>250 mM NaCl</td></tr>
<tr><td>3.</td><td>750 mM NaCl</td><td>6.</td><td>750 mM NaCl</td></tr>
</table>

**Table S3.** List of the primers used in the research.

| Primer name | Nucleotide sequences (5´-3´) | Primer designation |
|---|---|---|
| SaCLCa2R | GCCCTCAAATCTTGCCTT | Amplification of 3´- and 5´-ends *SaCLCa2* transcript sequences |
| SaCLCa2_5'RACE_R1 | GATCTTAACAAGCAATGTTGGTGCT | |
| SaCLCa2_5'RACE_R2 | GCCCCTGTCAACGTAATCAGC | |
| SaCLCa2_3'RACE_F1 | TCTTTGGGAGCATTGGAGC | |
| SaCLCa2F | TCCCGGAGAATTTCATCCAC | |
| pVR2_SaCLCa2_F | AAGGATCAAAACCATCATATGGAAGCAAGTGCGGAA TCACCA | Cloning of *SaCLCa2* into pVR2 vector |
| pVR2_SaCLCa2_R | ACTGACTCGACCCTATCATTTCTGCTTTTTGGATTTTGCT | |
| pMB1_SaCLCa2_F | ACACACATAAACAAACCATGGAAGCAAGTGCGGAATCACCA | Cloning of *SaCLCa2* into pMB1 vector |
| pMB1_SaCLCa2_R | ATCGATACCGTCGACCTCGAGTCATTTCTGCTTTTTGGATTTTGCTACA | |
| SaCLCa2_P182S_F | CCTACTGCTGCTGGGTCTGGTATCCCTG | Site-directed mutagenesis of *SaCLCa1(C544T)* |
| SaCLCa2_528_R | AGCAAAGAAAACAACCAGCAGCGTAG | |
| SaCLCc2F | TGTCCTCCTGAACTGCGAGA | Amplification of 3´- and 5´-ends *SaCLCc2* transcript sequences |
| SaCLCc2R | GCCTAATTCCCGAAACAACACT | |
| SaCLCc2_5'RACE_R1 | GTTGTAAACATGAGTGAGGCAAGATC | |
| pVR2_SaCLCc2_F | CGAAGGATCAAAACCATCATAGAGACATTTGAATAGCTGTGAGGTGA | Cloning of *SaCLCc2* into pVR2 vector |
| pVR2_SaCLCc2_R | CAGTACTGACTCGACCCTAAAGGGAAACTGCTCCTCAACATTGTA | |
| pMB1_SaCLCc2_F | ACACACATAAACAAACCATGGAAAATGAACTTTTTAAAGAGGATTGGA | Cloning of *SaCLCc2* into pMB1 vector |
| pMB1_SaCLCc2_R | ATCGATACCGTCGACCTCGAGCTACTTGTGGACTTCGAGGTGC | |
| SaClCa1_F1 | GAGACCTTGCTGAAGCCAAGC | RT-qPCR |
| SaClCa1_R1 | TGTCCCTTCACTAGTAAGGATTG | |

| | | |
|---|---|---|
| SaCLCa2_F1 | GTCCCAGAAAGACAAGGCACG | |
| SaClCa2_R1 | CCCATGTAAACTTCTCTCGCAC | |
| SaClCc1_F1 | GGCAAAGTATTTCTCTGAGGAG | |
| SaClCc1_R1 | AGATATATCCTCCAATGTGAGCC | |
| SaClCc2_F1 | CGACCAAATTGTTAAGATGAAGG | |
| SaClCc2_R1 | TGATCGTCCCAACTCTTTCAAC | |
| SaClCd_F1 | GGCTTCCCTGTGATAGATC | |
| SaClCd_R1 | CACCTCTTGAATCACTAGGC | |
| SaClCg_F1 | GGCTCCATGAGAATGACT | |
| SaClCg_R1 | TGAAACCATCAGCCACG | |
| SaClCf_F1 | GAAATGCTGCTGTTGCC | |
| SaClCf_R1 | CAGAAGCAGAACAGATGTCA | |
| SaAct7_F1 | AGATTCCGTTGCCCAG | |
| SaAct7_R1 | ATTTCCTTGCTCATACGGTCA | |
| SaeEF1alfa_F1 | TGAGATGTGTGGCAATCC | |
| SaeEF1alfa_R1 | GTTGCTTCTGACTCCAAGAAT | |
| pMB1_F | CATGGTTTGTTTATGTGTGTTTATTC | Amplification of a linear form of pMB1 vector |
| pMB1_R | CTCGAGGTCGACGGTATCGATAAGC | |
